# Identification of distinct transcriptome signatures of human adipose tissue from fifteen depots

**DOI:** 10.1038/s41431-020-0681-1

**Published:** 2020-07-13

**Authors:** Dorit Schleinitz, Kerstin Krause, Tobias Wohland, Claudia Gebhardt, Nicolas Linder, Michael Stumvoll, Matthias Blüher, Ingo Bechmann, Peter Kovacs, Martin Gericke, Anke Tönjes

**Affiliations:** 1 Department of Internal Medicine (Endocrinology and Nephrology), University of Leipzig, Leipzig, Germany; 2 IFB AdiposityDiseases, Leipzig University Medical Center, Leipzig, Germany; 3 Department of Diagnostic and Interventional Radiology, University of Leipzig, Leipzig, Germany; 4 Institute of Anatomy, Faculty of Medicine, University of Leipzig, Leipzig, Germany; 5 Institute of Anatomy and Cell Biology, Martin-Luther University Halle-Wittenberg, Halle, Germany

**Keywords:** Gene expression, Microarray analysis

## Abstract

The functional and metabolic characteristics of specific adipose tissue (AT) depots seem to be determined by intrinsic mechanisms. We performed a comprehensive transcriptome profiling of human AT from distinct fat depots to unravel their unique features potentially explaining molecular mechanisms underlying AT distribution and their contribution to health and disease. Post-mortem AT samples of five body donors from 15 anatomical locations were collected. Global mRNA expression was measured by Illumina® Human HT-12 v4 Expression BeadChips. Data were validated using qPCR and Western Blot in a subset of ATs from seven additional body donors. Buccal and heel AT clearly separated from the “classical” subcutaneous AT depots, and perirenal and epicardial AT were distinct from visceral depots. Gene-set enrichment analyses pointed to an inflammatory environment and insulin resistance particularly in the carotid sheath AT depot. Moreover, the epicardial fat transcriptome was enriched for genes involved in extracellular matrix remodeling, inflammation, immune signaling, coagulation, thrombosis, beigeing, and apoptosis. Interestingly, a striking downregulation of the expression of leptin receptor was found in AT from heel compared with all other AT depots. The distinct gene expression patterns are likely to define fat depot specific AT functions in metabolism, energy storage, immunity, body insulation or as cushions. Improved knowledge of the gene expression profiles of various fat depots may strongly benefit studies aimed at better understanding of the genetics and the pathophysiology of obesity and adverse body fat composition.

## Introduction

**Fig. 1:**
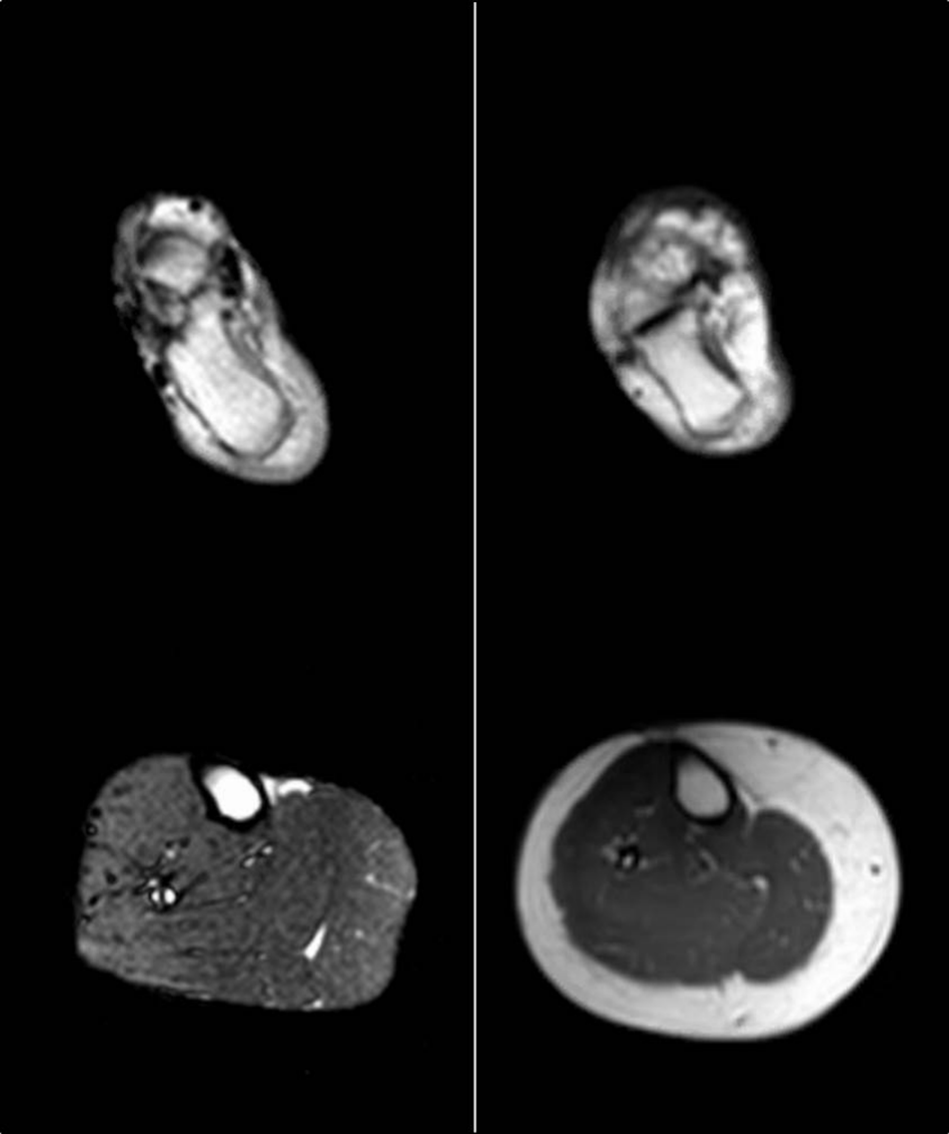
Magnetic resonance (MR) images of a sample patient and a healthy control. MR imaging using as a standard T1 sequence shows the right heel (upper row) and proximal calf (lower row) of a female patient with lipodystrophy (left column, BMI 27 kg/m²) and a female control (right, BMI 31 kg/m²). Written consent for publication of MR images was obtained from both subjects.

Adipose tissue (AT) is mainly composed of adipocytes and adipocyte precursor cells but also cells of connective tissue, vasculature, cells of the immune system, and nerve cells [1]. It does not only serve to store energy (mainly in white adipose tissue [WAT]) and to regulate body temperature, energy, and glucose balance (e.g., in brown adipose tissue [BAT], “beige” adipocytes in WAT) but also has mechanical functions (hand, heel, and renal capsule). Over the last decades AT has been treated as an endocrine organ [2, 3] supposed to secrete hundreds of bioactive factors contributing for example to the regulation of hunger and satiety, insulin sensitivity, inflammation, or ectopic lipid storage [4]. Fat distribution is controlled by genetic factors as suggested by numerous heritability studies [5]. However, the specific molecular mechanisms underlying AT distribution are still poorly understood. Extreme phenotypes of body fat distribution, i.e., steatopygia, lipodystrophies, or lipomatosis, are good models to address the genetic background of body fat distribution. In conditions of adverse AT distribution such as in lipodystrophies (e.g., familial partial lipodystrophy Dunnigan type (FPLD2, OMIM151660); congenital generalized lipodystrophy) certain AT depots are missing while others remain unaffected or even accumulate more fat [6]. For example in subjects with lipodystrophy type Dunnigan, the heel fat pad is the only location not affected by the extreme loss of subcutaneous adipose tissue (ScAT) which is observed at the limbs suggesting different pathways of lipid accumulation (personal observation, Fig. 1). Adipose progenitor cells and preadipocytes in different AT locations may derive from different cell lineages that determine their capacity for proliferation and differentiation and are influenced by the cell (micro-)environment, e.g., sex hormones or glucocorticoids, and the cellular composition of the depot as well as the extracellular matrix [7, 8]. As a result, rates of hypertrophy and hyperplasia during growth and remodeling vary among fat depots [7]. Lineage tracing studies raise evidence that white adipocytes may derive from different precursors such as mesenchymal progenitors (lateral plate mesoderm) and neural crest stem cells (ectoderm, suggested to be a source of ScAT) [9–13]. This is supported by transcriptional profiling studies that revealed characteristic expression pattern of developmental genes for subcutaneous and visceral WAT [7, 14, 15].

However, majority of these studies are restricted to the comparison of human AT depots which are accessible during surgery or biopsy in a defined body compartment. Body donors help to overcome this obstacle by providing a unique possibility to get access to adipose depots, which are regularly not available. In this study, we performed a comprehensive transcriptome profiling of human AT from 15 AT locations dispersed throughout the human body to unravel their unique features potentially explaining molecular mechanisms underlying AT distribution and their contribution to health and disease.

## Subjects, methods, data processing and statistical analysis

### Subjects

**Fig. 2:**
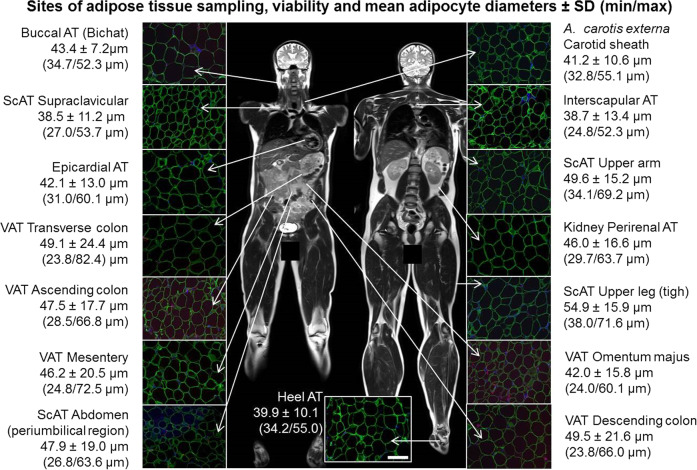
Sites of adipose tissue sampling, viability, and mean adipocyte diameters. Tissue slices of 7 µm were stained using antibody against the adipocyte specific marker perilipin (green), the macrophage marker AIF1 (red), and the nuclei were stained with DAPI (blue). Scale bar in the heel image represents 50 µm. For a larger view of the tissue slices see Supplemental Fig. 1). MR imaging was done with a male volunteer and written consent for publication of MR images was obtained. Mean adipocyte diameters ± SD (min/max) were calculated from measures of four body donors (1–3, and 6) with a post-mortem delay ≤12 h.

Human AT samples from 15 different fat depots (Fig. 2) were obtained from five body donors of the Institute of Anatomy at Leipzig University. Epicardial AT was available only for three body donors, and transverse colon was missing for one body donor. In total, 72 AT samples were initially available. During their lifetime, body donors gave their informed and written consent to the donation of their bodies for teaching and research purposes. As part of the body donor program regulated by the Saxonian Death and Funeral Act of 1994 (third section, paragraph 18 item 8), institutional approval for the use of the post-mortem tissues of human body donors was obtained from the Institute of Anatomy at Leipzig University. Samples were either snap frozen in liquid nitrogen for protein or RNA analyses or immediately fixed in 4% paraformaldehyde (PFA) and embedded in paraffin for histological evaluation. Additional characteristics including age, causes of death and post-mortem delay (pmd) are given in Table 1. For validation of microarrays RNA was extracted from seven further body donors (subcutaneous depots (periumbilical region, upper leg, and upper arm) as well as from heel fat pad; Table 1).

**Table 1:** Characteristics of the body donors.

Body donor ID	Gender	Age [years]	BMI [kg/m^2^]	Post- mortem delay [h]	Cause of death	Diagnoses	Purpose
1	Female	89	15.2	10	Pneumonia	–	Microarray; Histology; Western blots; qPCR
2	Male	64	23.4	10	Esophagus cancer	Cachexia, T2D	Microarray; Histology; Western blots; qPCR
3	Male	82	20.3	12	Heart attack	Heart failure	Microarray; Histology; Western blots; qPCR
4	Female	91	24.8	14	Heart failure	Hypertension, CHD, T2D, valvular aortic stenosis, stroke	Microarray
5	Female	98	30.3	24	Rectum cancer	Vascular encephalopathy, breast cancer, hypertension	Microarray
6	Male	78	33.2	12	Renal failure	Pneumonia; Alzheimers disease	qPCR; Histology
7	Female	87	22.2	19	COPD	Respiratory failure; Dementia	qPCR
8	Female	88	39.6	24	Leukemia	Hypertension	qPCR
9	Female	82	27.9	48	Respiratory failure	Hypertension; T2D; Heart failure	qPCR
10	Male	82	21.3	24	Stroke	Hypertension; COPD; Dementia; Prostatic cancer	qPCR
11	Female	81	28.4	24	Heart attack	Hypertension; T2D	qPCR
12	Female	87	23.6	24	Heart attack	Hepatocellular carcinoma	qPCR

*BMI* body mass index, *CHD* coronary heart disease, *T2D* type 2 diabetes mellitus, *COPD* chronic obstructive pulmonary disease, *qPCR* quantitative polymerase chain reaction.

## Methods

### Immunofluorescence staining of AT sections, measurement of adipocyte size, and Western Blot analysis

Immunofluorescence staining was done as previously described [16]. Mean adipocyte diameter has been assessed on H&E stained paraffin sections as described previously for the ATs of four body donors (donor 1–3, and 6; Table 1) [16, 17]. Western blot analysis was performed as described previously (Supplemental Material) [18].

### RNA extraction and cDNA synthesis

AT samples (100–130 mg) were homogenized in ball mill tubes (ceramic balls, 1.4 mm) in 1 ml Trizol® (Invitrogen-TFS, Germany; Supplemental Material). RNA was extracted using the InviTrap® Spin Tissue RNA Mini Kit (Stratec Molecular GmbH, Berlin, Germany) according to the manufacturer’s protocol “RNA “clean up” from Trizol® aqueous phase”. RNA integrity (RIN) and concentration were examined using an Agilent 2100 Bioanalyzer (Agilent, Santa Clara, California, USA). 1 μg of RNA per sample was reverse transcribed (Promega, Mannheim, Germany).

### Quantitative real-time RT-PCR analysis

For quantification of mRNA expression of the leptin receptor, quantitative real-time PCR was performed. Two different types of the leptin receptor were quantified: LEPR common (OB-R), detecting all forms of the leptin receptor, and LEPR full length (OB-Rfl), detecting only the long form (for primer sequences see Supplemental Material). The mRNA expression of the LEPR was determined relative to the 18S ribosomal RNA (18s) reference gene.

### Gene expression analyses using gene chips

mRNA expression data were obtained by Human HT-12 v4 Expression BeadChips (Illumina®, San Diego, California, USA). For the first three body donors tissues were processed in duplicates (excluding the ascending colon AT sample of body donor 1 due to the lowest RIN), and for upper leg each in quadruplicate. AT samples from body donor 4 and 5 were processed once.

### Data processing

#### Preprocessing

Raw data of 47,323 gene-expression probes and 887 control probes were extracted by Illumina GenomeStudio without additional background correction. The data were further processed within R/Bioconductor R [19]. Initially, six samples were excluded: (i) three samples (2.5%) because of low RNA quality, and (ii) three samples (2.5%) having an extreme number of expressed genes (defined as median Â± 4 × interquartile ranges (IQR) of the cohort’s values). Transcripts not expressed at *p* = 0.05 (as defined by Illumina and implemented in the R/Bioconductor package lumi [20]) in at least 5% of all samples per tissue location were excluded from further analysis. 37,928 (80.1%) probes remained in the analysis in all subgroups. Expression values were quantile-normalized and log_2_-transformed [21]. For an extended description please see the Supplemental Material.

#### Outlier detection and batch-correction

We adapted an approach from Oldham et al. [22]. This was done separately for all subgroups and resulted in 11 outlier samples (9.1%). We used a linear approach for batch-correction. We found the pmd and the Sentrix barcode as best fitting batch effect model and corrected for these effects. For further outlier detection, we calculated the Euclidian distance between all samples of the same subgroup and the group-center. This was defined as the average of samples after removing 10% of samples manifesting largest distances from the group-center done separately for each subgroup (implemented in the R/Bioconductor package lumi [20]). The threshold for outlier detection was defined as two times the median distance to the center.

#### Gene mapping

Mapping of genes corresponding to expression probes and assignment of gene names was done using information of a remapping approach [23] applying gene-information of the NCBI database. This information was retrieved using the R add-on package from Bioconductor illuminaHumanv4.db_1.26.0 that relates to NCBI data dated on 2015-March-17. This resulted in a total of 28,430 valid gene-expression probes (=variables) corresponding to 20,213 unique genes available in all subgroups.

#### Visualization

We used the Qlucore Omics Explorer (QOE, Qlucore, Lund, Sweden) for visualization of the data and the statistical analyses. Predicted loci were checked in the actual version of the NCBI database [GRCh38, last access May 2019] and if available, the gene was assigned or the probe was withdrawn from the list count.

#### Data statement

The dataset used for the analyses with Qlucore was submitted to figshare (https://figshare.com/about) and is accessible via the DOI 10.6084/m9.figshare.11836032 or the following link:

https://figshare.com/articles/Identification_of_distinct_transcriptome_signatures_of_human_adipose_tissue_from_fifteen_depots_/11836032.

### Statistical analysis

Mean values were calculated for duplicates/quadruplicates. Finally, 54 samples were included in the analysis (N for each depot see Supplemental Material). Sigma-normalization was applied to the dataset. The dataset was not pre-collapsed due to the assignment of the same gene symbol to different transcripts represented by different probes. Principal component analyses and hierarchical clustering were performed to investigate the structure of the data. Multi-group and two-group comparisons between the AT depots were performed using the *F*-test (ANOVA) and Student’s *t* test (two-sided), respectively. The analyses were adjusted for age and gender. A *p* value <1.1 × 10^−6^ was defined to be significant. The DAVID 6.8 Functional Annotation Tool (https://david.ncifcrf.gov/) and the Gene Ontology enRIchment anaLysis and visuaLizAtion tool “GOrilla” (http://cbl-gorilla.cs.technion.ac.il/) were used for enrichment analysis and visualization [24, 25].

## Results

### Adipocyte size, viability of AT from body donors, and evaluation of RNA quality for microarray analysis

**Fig. 3:**
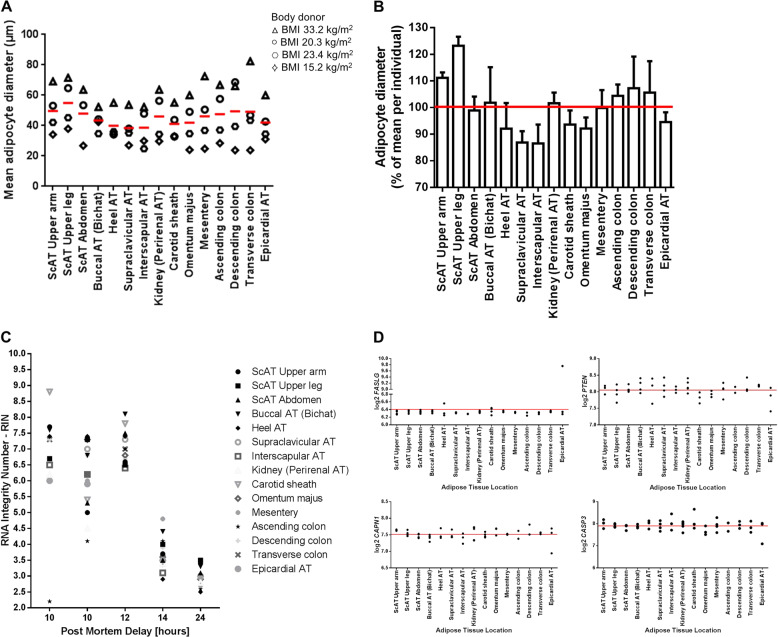
Adipocyte characteristics. **a** Mean adipocyte diameter in µm for four body donors with a pmd ≤ 12 h. **b** Mean adipocyte diameter in % of mean per individual. **c** RNA Integrity Number (RIN) plotted against post-mortem delay (pmd) for each body donor sampled on microarrays. RNA quality decreases with increasing pmd. Each column represents one body donor. **d** Marker for autolysis. Given are the mRNA expression values (log2 transformed) for each sample represented by a dot. The continuous line represents the mean overall samples. The data are adjusted for post-mortem delay, Sentrix barcode, age and sex. FASL-Fas ligand, PTEN-phosphatase and tensin homolog, CAPN1-calpain 1, CASP3-caspase 3.

Adipocyte diameters were distributed according to the BMI of the donors (Fig. 3a). Interestingly, adipocytes from VAT and ScAT omentum majus show lower adipocyte diameters in comparison to all other investigated visceral AT (mesentery, colon AT samples) and ScAT depots respectively (Fig. 3b). AT sections of all 15 studied fat depots revealed a high degree of perilipin expressing adipocytes indicating high tissue viability (Fig. 2, Supplemental Fig. 1). AIF1 expressing AT macrophages were also present in most of the sections, but characteristic crown-like structures (typically found around dying adipocytes) were lacking (Fig. 2, Supplemental Fig. 1). We observed a broad spectrum of RINs for the tissues (RINs ranging from 2.2 to 8.8, median 6; Fig. 3c). After preprocessing of the data the following tissues were included in the analysis: all depots for body donors 1–3 except for VAT ascending colon of body donor 1; carotid sheath, VAT mesentery, perirenal AT, ScAT abdomen of body donor 4; and perirenal AT, ScAT abdomen, ScAT upper arm, ScAT upper leg, supraclavicular AT and buccal AT from body donor 5. In addition, the analysis of the mRNA expression of markers of autolysis (*FASL*, *PTEN*, *CAPN1*, and *CASP3*) did not reveal significant differences between the tissues (Fig. 3d).

### Characterization of AT depot-specific gene expression signatures

**Fig. 4:**
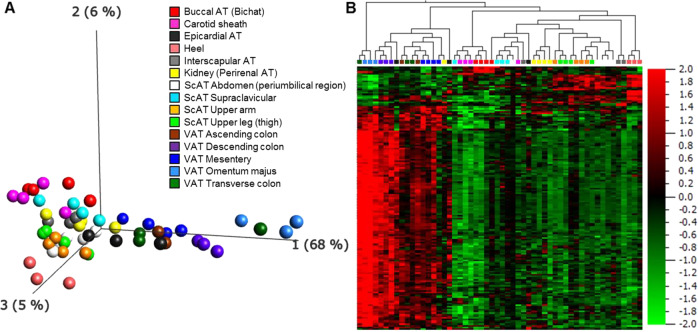
Adipose tissue depot-specific gene expression signatures. Principal Component Analysis (**a**) and hierarchical clustered heat map (**b**) of 15 AT depots in the multigroup comparison. A *p* value of 1.1 × 10^−6^ (genome-wide significance level of the study) was applied resulting in 165 remaining transcripts.

To explore the overall gene expression differences between the 15 different AT locations, the preprocessed dataset was analyzed with *F*-test (ANOVA) using the location of the tissue samples as grouping variable. At *p* < 1.1 × 10^−6^ 68% of the variance were explained by the first principal component with 165 variables (which can be collapsed to 132 validated genes) remaining in the analysis (Fig. 4a; Supplementary document – Table 1). ATs from the body cavities (except for kidney) present a clearly distinct expression pattern as compared with the AT from all other locations (Fig. 4b). Of note, the epicardial AT clusters with the visceral AT depots from the abdomen and the kidney AT clusters with the ScAT samples. Whereas the body cavity depots, except for kidney, manifest a homogeneous expression pattern, the expression pattern is more diverse in subcutaneous depots with distinct signatures for buccal, heel, and carotid sheath AT. In a separate analysis of gene expression of selected genes in tissues of two body donors with a pmd of 10 h, buccal, heel, and supraclavicular AT displayed higher expression of genes involved in adipogenesis and lipolysis compared with the other tissues (Supplementary Fig. 2). The samples of one tissue location were compared against all other tissue locations grouped together to look for gene sets specific for this fat tissue (differential expressed transcripts with *p* ≤ 0.001). We identified significant GO terms for most of the subcutaneous locations and the VAT omentum majus as well as VAT mesentery area suggesting that the expression pattern of the AT depot reflect their location (Supplementary Fig. 3; “Gene Lists for GOrilla” in the Variable_Lists_collection_V1-6_Supplemental document_1). However, one need to keep in mind that in the respective analyses only a maximum of five samples were compared against all other samples (*N* = 49) which clearly limited their statistical power. In addition, we investigated the AT depots of the body donors for signs of beiging/browning (Supplementary Results, Supplementary Fig. 4).

### Discriminant analysis for identification of major determinants of variability

To identify major determinants of variability, AT depots (sub-locations) were organized in groups based on the anatomical location (Fig. 2) and based on the results of the multigroup comparison.

#### Comparison of visceral- vs. subcutaneous-like AT depots

The depots from the body cavities (VAT samples, epicardial AT) were compared with the “subcutaneous-like” depots (ScAT samples, in addition AT from buccal, heel, interscapular, carotid sheath, and including the kidney samples) in a two-group comparison. At *p* < 1.1 × 10^−6^ 347 variables separate both groups corresponding to 275 genomic loci (collapsed for gene ID as a unique identifier, including confirmed predicted annotations, excluding non-specified variables; Supplementary document - Table 2). Twenty-one genes were higher expressed in the subcutaneous-like tissues, including developmental genes such as *IRX3*, *TBX15*, *PRRX1,* or *SIX1* (Supplementary document - Table 2). Among the 271 transcripts (corresponding to 250 genes) found to be higher expressed in the visceral depots are mostly represented by genes that are described to play a role in the insulin pathway, in adipogenesis, and in obesity-associated inflammation (Supplementary document – Table 2). Mapping by DAVID indicated enrichment of genes involved in organ and tissue morphogenesis (Gene Ontology term for biological processes (BP)_FAT GO:0009887, GO:0048729; *p* < 1.9 × 10^−6^, False Discovery Rate (FDR) < 0.005; Supplementary document – Table 2.1).

#### Inter-depot comparison: visceral depots

In the next step, we compared gene expression patterns of different AT sub-depots of the intra-abdominal and intra-thoracic cavities (epicardial, ascending colon, descending colon, transverse colon, mesentery, and omentum majus).

**Fig. 5:**
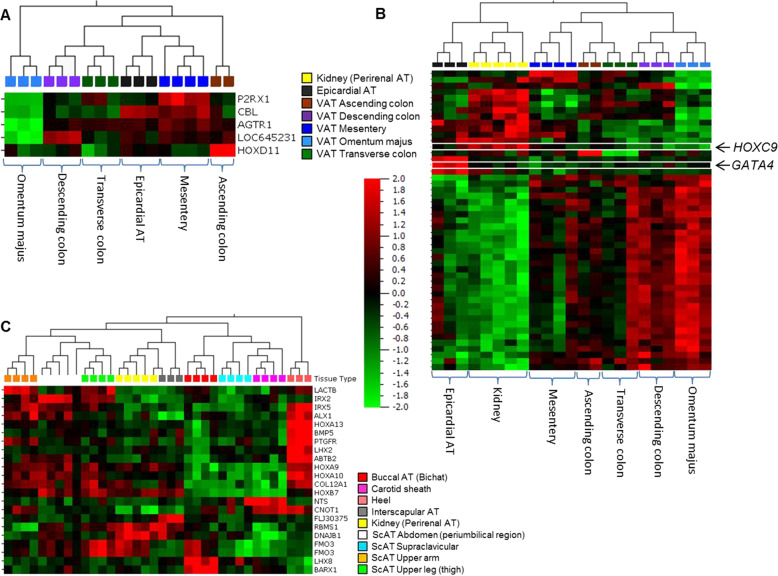
Discriminant analysis for identification of major determinants of variability between AT samples of different locations in the body. Hierarchical clustered heat maps of (**a**) samples derived from the body cavity. *F*-test, *p* < 0.001 and FDR-threshold 0.2. **b** Samples from the body cavity including kidney. *F*-test, *p* < 0.001 and FDR-threshold 0.2. **c** Samples from all subcutaneous locations, and buccal, heel, carotis sheath and kidney. *F*-test, *p* < 1.1 × 10^−6^. For further data please see Supplementary document Table 3.

At *p* < 1.1 × 10^−6^ only *angiotensin II receptor type 1* (*AGTR1*) gene expression was lower in the VAT omentum majus samples compared with the other locations. Applying a *p* value of <0.001 and a FDR-threshold of 20% resulted in five loci of which four have been assigned to a gene (Fig. 5a). Interestingly, perirenal and epicardial AT are clearly distinct from visceral depot samples characterized e.g., by the expression of *HOXC9* in perirenal, and *GATA4* in epicardial AT (Fig. 5b, Supplementary document – Table 3).

#### Inter-depot comparison: subcutaneous depots and perirenal AT

Comparisons of the subcutaneous ATs from upper leg, upper arm, abdomen, interscapular, and supraclavicular as well as the locations from buccal, heel, carotid sheath, and also kidney revealed that AT gene expression signatures from buccal, heel, and carotid sheath deviated significantly (*p* < 1.1 × 10^−6^) from all other ScAT depots (Fig. 5c, Supplementary document – Table 3). The tissues are characterized by higher expression of homeobox genes (Fig. 5c). After excluding buccal, heel, and carotid sheath AT from the analysis, remaining ScATs and perirenal AT were not significantly different in gene expression at genome-wide significance level. However, at *p* < 0.001 and FDR ≤ 20% 93 variables corresponding to 74 genes cluster the samples, separating kidney, interscapular, and supraclavicular AT from the ScAT depots of upper arm, upper leg, and abdomen (Supplementary document – Table 4).

#### Comparative analysis of AT depots from buccal, heel, carotid sheath, and epicardial AT

Subcutaneous depots from abdomen and extremities were merged to a group “subcutaneous”. To identify markers distinguishing the AT depot from buccal, heel, and carotid sheath from the subcutaneous depots two-group comparisons were performed (subcutaneous vs. buccal, heel or carotid sheath, respectively).

**Fig. 6:**
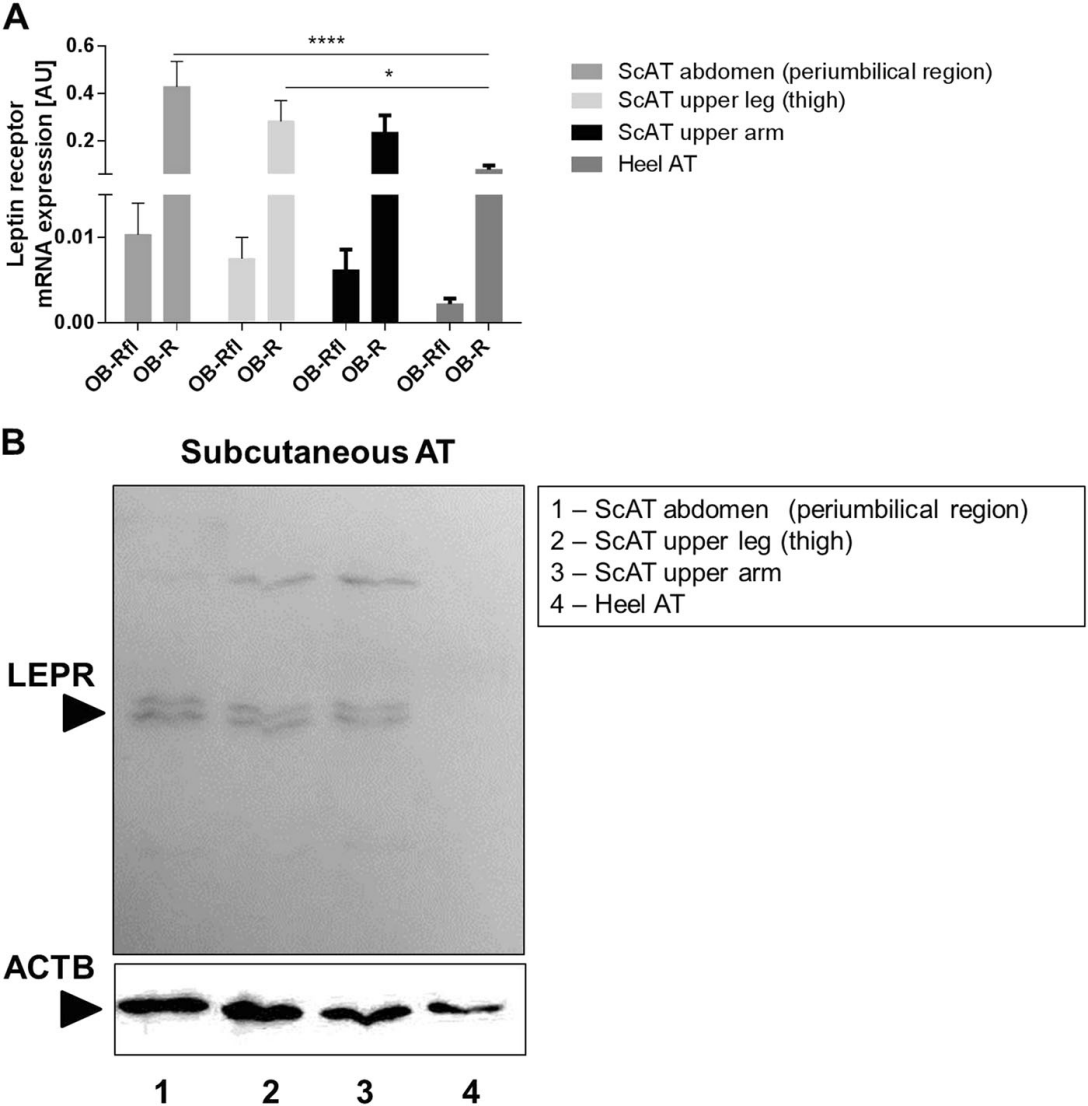
LEPR mRNA and protein expression in subcutaneous AT from body donors. **a** *LEPR* mRNA expression is significantly lower in heel AT compared with other subcutaneous AT locations. Ob-Rfl—*LEPR* full length, detecting only the long form of the receptor; Ob-R—all forms of the leptin receptor are detected. ****/*p < 0.0001/0.05. **b** Protein expression: LEPR is expressed in subcutaneous AT but not detectable in heel AT. Multiple bands occur due to different isoforms of the receptor.

First, the subcutaneous depots were compared with the buccal samples. The observed differences, revealed 44 validated transcripts corresponding to 42 genes (*p* ≤ 7.7 × 10^−5^, FDR ≤ 5%) of which 22 are higher expressed in the buccal depot (Supplementary document – Table 5). Among these transcripts are homeobox genes and transcription factors that are described to be involved e.g., in tooth morphogenesis and development (*LHX8*, *BARX1*, and *PAX9*). Second, the depot from heel was compared with the subcutaneous samples. Three transcripts are significantly higher expressed in the AT samples from heel: *HOXA13*, *LHX2*, and *ALX3*. Applying a less stringent *p* value of ≤0.001 resulted in 74 differentially expressed validated transcripts, which correspond to 71 genomic loci (Supplementary document – Table 6) with gene enrichment in embryonic morphogenesis and limb development (Supplementary document – Table 6.1). Interestingly, in the heel depot the *LEPR* is expressed at very low levels. However, in this dataset FDR was high (33.3%). We confirmed the reduced *LEPR* expression in heel AT in comparison to subcutaneous abdominal and thigh AT by quantitative real-time PCR (*p* < 0.0001; <0.05, respectively; Fig. 6a, b).

Third, gene expression from the fat depot of the carotid sheath was compared with the subcutaneous depots. Three loci were higher expressed (*p* < 1.1 × 10^−6^), *MAL*, *NTS*, *PKIA*; and *CLTA* was lower expressed. Applying a FDR of ≤5% resulted in 266 differential expressed variables (*p* ≤ 4.65 × 10^−4^) corresponding to 241 valid transcripts and 234 genomic loci (Supplementary document – Table 7). This depot is characterized by the enrichment of genes involved in biological processes related to the immune system which points to the fact that this tissue may have had exhibited inflammatory milieu in the body donors (Supplementary document – Table 7.1).

Likewise, epicardial AT samples were compared with visceral depots (omentum majus, mesentery, ascending colon, descending colon, and transverse colon) clustered in the term “visceral” (two-group comparison). At *p* = 1.1 × 10^−6^ two transcripts were higher expressed in the epicardial depot compared with the visceral samples, *COL4A4* and *HBM*. In a less stringent analysis using a *p* value of ≤0.001 and a FDR < 20% 99 differential expressed valid transcripts (corresponding to 98 genes) are displayed (Supplementary document – Table 8), which included genes involved in myocardial differentiation and function.

## Discussion

Here, we compare AT gene expression signatures originating from 15 distinct fat depots of human body donors. It has been previously shown that the quality of RNA is highly tissue, donor, and pmd dependent [26, 27]. The influence of the pmd was also obvious in our AT samples as we had to exclude some AT samples from two body donors with pmd of 14 and 24 h, respectively. The multigroup comparisons of all 15 depots revealed that the main difference between the locations is derived from samples grouped in the general term “VAT” (including the epicardial AT) vs. those grouped as “ScAT” (including buccal, carotid sheath, heel, kidney, and interscapular AT). Interestingly, the kidney samples are clearly separated from the VAT samples and located in the ScAT group. In line with previous studies [28, 29], depot specific expression of developmental genes like the *HOX family* was observed in our study as well. Although the well acknowledged depot specificity of certain *HOX* genes is evident, the *HOX* expression pattern seems to be highly dependent on the specific tissues clustering and individual pair-wise comparisons (see Supplementary document Tables). Also consistent with previous reports, we confirmed differences between ScAT and VAT in gene expression patterns including not only developmental genes, but also genes potentially contributing to aberrant adipocyte function during obesity such as *SIX1*, which binds to adipogenic and brown marker genes and interacts with *C/EBPα*, *C/EBPβ*, and *EBF2* [30].

### Facets of epicardial and perirenal AT

There is strong evidence that epicardial AT plays an important role in heart disease and that there is a cross-talk between epicardial fat and the heart [31]. The epicardial fat transcriptome shows an enrichment of genes involved in extracellular matrix remodeling, inflammation, immune signaling, coagulation, thrombosis, beigeing, and apoptosis (compared with ScAT) [32]. Consistently, in epicardial AT we found significantly higher expression of genes representing these pathways (e.g., *COL4A4*, *HBM*, *GATA4*, amd *CD68*). Regulation of angiogenesis (GO:0045765) and regulation of vasculature development (GO: 901342) turned out to be the two major GO-terms. Comparing the epicardial AT mRNA expression with ScAT as previously reported by Gaborit et al. [32], we confirmed *omentin* (*ITLN1*) to be the strongest differentially expressed gene in the dataset (*p* = 9.78 × 10^−9^; data not shown). Omentin is an adipokine known to exert beneficial effects on metabolism [33] and to play a role in the pathogenesis of coronary artery disease [34].

Perirenal fat was described to be a predictor of early kidney damage in non-hypertensive and non-diabetic obese patients [35] and perirenal fat invasion seems to be a critical factor in renal cell carcinoma [36]. In our dataset, the kidney fat appears more distinguishable from the VAT than ScAT. Although the expression pattern in perirenal AT is more similar to the pattern in ScAT, it can still be separated according to differences in the expression of genes such as *ankyrin repeat domain 20 family member A1* (*ANKRD20A1*), *RNA binding motif single stranded interacting protein 1* (*RBMS1*), and *DnaJ heat shock protein family (Hsp40) member B1* (*DNAJB1*) (data not shown). Of note, we did not detect any features of browning in perirenal AT samples but this might be due to the high individual variability in the tissue as described by Svensson et al. [37].

### Facets of buccal, heel, interscapular, supraclavicular, and carotid sheath AT

The AT depots of buccal and heel were clearly separated from other the ScAT depots. Buccal AT is not only important for mastication and sucking in neonates and as cushion to protect neurovascular bundles but it also plays a role in oncological defects and in aesthetic facial surgery [38]. Interestingly, *LHX8*, which was previously described as a BAT-selective marker [39], was characteristic for the buccal AT depot. However, histologically it was described as pure white fat with less vesicles and smaller mitochondria indicative of a tissue manifesting lower metabolic activity [40]. *LHX8* as well as other genes expressed in buccal AT (*BARX1*, *PAX9*) have been implicated to play a role in tooth morphogenesis in mice and men [41, 42]. The heel AT has been investigated for fatty acid composition compared with subcutaneous abdominal fat so far [43] but we are not aware of any gene expression study. Here, we found higher mRNA expression of genes that are involved in embryonic and limb morphogenesis and skeletal system development (*HOXA13*, *LHX2*, *COBL*, *HOXA11*, *SALL1*, *TBX4*, *ALX3*, *PITX1*, and *BMP5*). The heel fat pad is for example affected in conditions of rheumatoid arthritis or peripheral neuropathy [44, 45]. In congenital generalized lipodystrophy, mechanical AT depots like the heel fat pad remain unaffected [6] which might be due to a different metabolic program. For instance, the *LEPR* showed very low expression in heel AT and could not be detected on protein level. The samples from the carotid sheath showed an enriched expression of genes described to play a role in insulin resistance (*PPP1R3E*, *PRKCQ*, *PRKCZ*, *CPT1B*, *MGEA5*, *RPS6KB2*, *OGT*; KEGG_PATHWAY hsa04931:Insulin resistance). Furthermore, *NTS* was significantly higher expressed in the carotid sheath AT samples. NTS is distributed in the central nervous system, is involved in the maintenance of gut structure and function, and in the regulation of fat metabolism [46]. Besides the suggested involvement in cancer [47] NTS was recently described to play a role in diet-induced obesity [48]. In the Framingham Heart Study, proneurotensin concentrations were positively associated with the risk of incident cardiovascular events [49]. Neurotensin seems to affect the coronary vascular tone in the rat, guinea-pig and dog, and the gastric mucosal blood flow in humans [50] and the same might be true for the carotid artery.

### Study limitations

We have to acknowledge several limitations of the study: the sample size is small, whole AT samples including several cell types have been used for the analysis of the transcriptome, the age range of the donors was between 64 and 98, and comorbidities and causes of death differed between the subjects, which might have influenced the expression profiles. Nevertheless, studies offering the possibility of intra-individual comparisons of 15 AT depots derived from the same body donor are lacking, which makes the here presented design unique.

## Conclusion

The expression pattern of the AT depots strongly reflects their location. The study provides a comprehensive view on the human AT depots to improve our knowledge of the gene expression profiles of various fat depots, which may strongly benefit studies aimed at better understanding of the genetics and the pathophysiology of adverse body fat composition.

## Supplementary information

Supplemental Material

Supplemental Figure 1: **Viablility of AT from body donors.** Tissue slices of 7 µm were stained using antibody against the adipocyte specific marker perilipin (green), the macrophage marker AIF1 (red), and the nuclei were stained with DAPI (blue). Scale bar represents 50 µm.

Supplemental Figure 2: **Proportion of gene expression between the tissues of selected biological functions and pathways.** Calculation in percent based on the mean tissue expression of two body donors with a post mortem delay of 10 hours. White-blue-red coding=0-100%. *(For readability, please open the figure with other software than Wndows photo.)*

Supplemental Figure 3: **Top enriched GO term for process (1), function (2) & component (3).** Boxes for the respective tissue display top enriched GO term with p<0.05 and false discovery rate (FDR) q-value<0.2. 1) process (p-value, FDR q-value, enrichment) and/or 2) function (p-value, FDR q-value, enrichment) and/or 3) component (p-value, FDR q-value, enrichment). Gene lists from the single tissue comparison against all other tissues from the visceral area including epicardial AT were also combined for GOrilla as the single information for these tissues would not withstand statistical significance with the respective cut off for the false discovery rate (dashed line box). Analyzed transcripts for this figure are given in “Gene Lists for GOrilla” in the Variable Lists collection V1-6 Supplemental document 1.

Supplemental Figure 4: **Markers for beiging/browning.** Given are the mRNA expression values for each sample represented by a dot for the following markers: *BMP7*, *CIDEA*, *EBF2*, *FGF21*, *LHX8*, *PPARG*, *PRDM16*, *TBX1*, *TMEM26*, *UCP1*. The continuous line (red) represents the mean over all samples. The small lines (green) represent the mean for all samples from one tissue location. The data displayed here are pre-processed, log2 transformed and are adjusted for post mortem delay, Sentrix barcode, age and sex. Of note, the Y-axes have different scaling. Further investigated marker with similar results (data not shown): *ADAM17*, *BMP4*, *CAR4*, *TNFRSF9*, *CITED1*, *COX4*, *HOXC8/9*, *HSPB7*, *MIR133B*, *miR26-family*, *PPARGC1A*, *ZIC1*.

Variable Lists collection V1-6 Supplemental document 1
