# Supplementary material for: Identification of distinct transcriptome signatures of human adipose tissue from fifteen depots
Source: Eur J Hum Genet. 2020 Jul 13;28(12):1714–25. doi: 10.1038/s41431-020-0681-1 (PMC7784683; doi:10.1038/s41431-020-0681-1)
Supplement: Supplementary file 1 — Supplemental Material [file 41431_2020_681_MOESM1_ESM.docx]

**Supplemental Material**

**Table of Content**

1) Supplemental Methods

2) Supplemental Results and Discussion

3) Supplemental References

**1) Supplemental Methods**

*Immunofluorescence staining*

7 µm thick sections were cut, deparaffinized, and rehydrated. Antigen retrieval was performed using Tris/EDTA buffer (pH 9.5) for 2 x 5 min at 95°C. AT sections were rinsed three times in PBS with 0.3% Triton-X (PBST; Sigma- Aldrich, St Louis, Missouri, USA). Blocking of nonspecific binding sites was done by using 1% bovine serum albumin in PBS supplemented with 0.3% Triton-X for 30 min at RT (Staining buffer). Rabbit anti-AIF1 (allograft inflammatory factor 1; 1:500, Wako, Richmond, VA) and goat anti-perilipin A (1:200, Abcam, Cambridge, UK) were diluted in staining buffer and incubated overnight at 4°C. Donkey anti-goat Alexa 488 and donkey anti rabbit Alexa 568 (diluted at 1:200 in staining buffer; both from Invitrogen, Karlsruhe, Germany) were used as secondary antibodies (1 h at RT). Autofluorescence of the tissue was quenched by using 0.3% sudan black solution for 2 minutes. Nuclear counterstain was done using 4,6-diamidin-2-phenylindol (DAPI; 1:10,000) for 5 min. Finally, sections were embedded with Dako immunofluorescence mounting medium (DAKO, Glostrup, Denmark).

*Western blot analysis*

Proteins were extracted using RIPA buffer (50 mM Tris [pH 8], 150 mM NaCl, 1% Nonidet P-40, 0.5% sodium deoxycholate, and 0.1% SDS, supplemented with 1% PMSF and 1% protease inhibitor mixture [Sigma-Aldrich, Munich, Germany]). Protein concentration was assessed using the BCA protein assay (Pierbo Science, Bonn, Germany). Proteins (20 µg) were denatured for 5 min at 95°C, separated by 7.5% SDS-polyacrylamide gel electrophoresis and transferred to a nitrocellulose membrane. Nonspecific binding sites were blocked by pre-incubation with 5% non-fat milk for 30 min. Subsequently, blots were incubated with polyclonal antisera against the leptin receptor (ab5593; 1:1,000; Abcam, Cambridge, UK) or anti-b-Actin (3700; 1:1,000; Cell signaling, Danvers, MA, USA) at 4°C overnight. Immunoreactions were detected with the appropriate peroxidase-conjugated goat anti-rabbit IgG secondary antibodies (1:10,000; Vector Laboratories, Peterborough, U.K.) at RT for 1 h. Peroxidase activity was visualized with an ECL kit (Amersham Pharmacia, Freiburg, Germany).

*RNA extraction and cDNA synthesis*

AT samples of 100 to 130 mg were homogenized in ball mill tubes (ceramic balls, 1.4 mm) in 1 ml Trizol® (Invitrogen-TFS, Germany) using the tissue homogenizer Precellys® (VWR, Radnor, Pennsylvania, USA) for 2x 5 sec at 5500 rpm. The homogenate was transferred to a new tube and 200 µl chloroform-isoamyl alcohol was added to each sample. The samples were vortexed for 15 sec following a 3 min incubation step at room temperature, and a centrifugation step for 10 min at 4°C and 14,000 rpm. The aqueous phase was transferred to a new tube. RNA was extracted using the InviTrap® Spin Tissue RNA Mini Kit (Stratec Molecular GmbH, Berlin, Germany) according to the manufacturer’s protocol “RNA “clean up” from Trizol® aqueous phase”. RNA integrity (RIN) and concentration were examined using an Agilent 2100 Bioanalyzer (Agilent, Santa Clara, California, USA). 1 μg of RNA per sample was reverse transcribed in a final mixture of 5x first-strand buffer (250 mM Tris-HCl, pH 8.3, 375 mM KCl,15 mM MgCl 2 ), 0.5 mM of dNTPs, 5 mM of DTT (Promega, Madison, Wisconsin, USA), 15 U of Prime RNase Inhibitor (ThermoFisher, Waltham, Mass., USA), 0.5 μg of random hexamer primers and 200 U of Moloney murine leukemia virus reverse transcriptase (Promega, Mannheim, Germany). Reverse transcription was performed at 37°C for 60 min and 94°C for 5 min.

*Primer sequences for LEPR*

The following primers were used: OB-R: 5’-AAGAGGCTAGATGGACTGGGATATT-3’ (forward), 5’-ATTCTCCAAAATTCAGGTCCTCTCA-3’ (reverse); OB-Rfl: 5’-GTTCCTGGGCACAAGGACTTA-3’ (forward) and 5’-ACAGTTGTTGGCATCATCTCATC-3’ (reverse), and 18S 5’- GTAACCCGTTGAACCCCATT (forward) and 5’- CCATCCAATCGGTAGTAGCG – 3’ (reverse).

*Gene Expression Analysis using Gene Chips*

**Preprocessing**

Raw data of 47,323 gene-expression probes and 887 control probes were extracted by Illumina GenomeStudio without additional background correction. The data were further processed within R/Bioconductor R (ref. 19). Initially, three samples (2.5%) were excluded because of low RNA quality detected during sample preparation. Further, three samples (2.5%) having an extreme number of expressed genes (defined as median Â± 4 x interquartile ranges (IQR) of the cohort’s values) were excluded. Transcripts not expressed at p = 0.05 (as defined by Illumina and implemented in the R/Bioconductor package lumi (ref. 20)) in at least 5% of all samples per tissue location were excluded from further analysis. 37928 (80.1%) probes remained in the analysis in all subgroups. Expression values were quantile-normalized and log2-transformed (ref. 21).

**Outlier detection and batch-correction**

Further we adapted an approach from Oldham et al. (ref. 22). This was done separately for all subgroups and resulted in eleven outlier samples (9.1%).

Due to our relatively small subgroups we used a linear instead of an empirical Bayes approach for batch correction. We found the pmd and the Sentrix barcode as best fitting batch effect model and corrected for these effects. For further outlier detection, we calculated the Euclidian distance between all samples of the same subgroup and the group-center. This was defined as the average of samples after removing 10% of samples manifesting largest distances from the group-center done separately for each subgroup (implemented in the R / Bioconductor package lumi (ref. 20)). The threshold for outlier detection was defined as two times the median distance to the center.

**Gene mapping**

Mapping of genes corresponding to expression probes and assignment of gene names was done using information of a remapping approach (ref. 23) applying gene-information of the Entrez gene database of the National Center for Biotechnology Information (NCBI), available at http://www.ncbi.nlm.nih.gov/gene/. This information was retrieved using the R add-on package from Bioconductor illuminaHumanv4.db_1.26.0 that relates to NCBI data dated on 2015-March-17.

This remapping approach resulted in a total of 28,430 valid gene-expression probes (=variables) corresponding to 20,213 unique genes available in all subgroups.

**Visualization**

We used the Qlucore Omics Explorer (QOE, Qlucore, Lund, Sweden) for visualization of the data and the statistical analyses. Predicted loci were checked in the actual version of the NCBI database [GRCh38, last access May 2019] and if available the gene was assigned or the probe was withdrawn from the list count.

*Statistical Analysis*

Mean values were calculated for duplicates/quadruplicates. Altogether 54 samples included in the analysis (omentum majus: N=3; ascending colon: N=2; descending colon: N=3; transverse colon: N=3; mesentery: N=4; supraclavicular: N=4; upper arm N=4; abdomen: N=5; upper leg: N=4; heel: N=3; interscapular N=3; epicardial: N=3; carotid sheath: N=4; kidney: N=5; buccal: N=4). Sigma-normalization was applied to the data set (mean=0, var=1, correlation matrix). The dataset was not pre-collapsed due to the assignment of the same gene symbol to different transcripts represented by different probes. Principal component analyses (PCA) and hierarchical clustering were performed to investigate the structure of the data. Multi-group and two-group comparisons between the AT depots were performed using the F-test (ANOVA) and Student’s t-test (two-sided), respectively. The analyses were adjusted for age and gender. A p-value <1.1x10 6 was defined to be significant. The DAVID 6.8 Functional Annotation Tool was used for enrichment analysis (ref. 24).

**2) Supplemental Results and Discussion**

*Signatures of browning within white AT*

During the last decade strong evidence has emerged for the existence of brown adipocytes in adults supported by studies suggesting that adipocytes in white AT depots can be activated for a brown-like/beige phenotype (supplemental [suppl.] ref. 51-53). Morphologically, beige and brown adipocytes can be distinguished from white adipocytes as they contain multilocular lipid droplets and dense mitochondria (suppl. ref. 53). Beige cells have been found in nearly every AT depot (ref. 39; suppl. ref. 54-56) and a couple of gene expression markers such as *uncoupling protein 1* (*UCP1*) have been described to distinguish them from white adipocytes (suppl. ref. 57;58). We investigated the AT depots of the body donors for signs of beiging/browning histologically and checked the gene expression of beige/brown markers (*BMP7*, *CIDEA*, *EBF2*, *FGF21*, *LHX8*, *PPARG*, *PRDM16*, *TBX1*, *TMEM26*, *UCP1* and others (Supplemental Figure 4). Multilocular adipocytes (≤5) could be detected in one epicardial and one supraclavicular AT sample, however the analysis of the gene expression markers does not support the presence of beige/brown adipocytes in comparison to the other depots (Supplemental Figure 4). In conclusion, in the tissues we derived from the body donors we could not detect distinct signs of browning/beigeing on histological and molecular level which might be, at least partially due to the age of the donors as aging is negatively associated with the appearance of active brown fat in mice and men (suppl. ref. 59;60). Furthermore, there is no functional sympathetic innervation anymore post mortem.

**3) Supplemental References**

[**51**] Virtanen KA, Lidell ME, Orava J, Heglind M, Westergren R, Niemi T *et al.* Functional brown adipose tissue in healthy adults. *N Engl J Med.* 2009; **360:** 1518-1525.

[**52**] Nedergaard J, Bengtsson T, Cannon B. Unexpected evidence for active brown adipose tissue in adult humans. *Am J Physiol Endocrinol Metab.* 2007; **293:** E444-452.

[**53**] Wu J, Boström P, Sparks LM, Ye L, Choi JH, Giang AH *et al.* Beige adipocytes are a distinct type of thermogenic fat cell in mouse and human. *Cell.* 2012; **150:** 366-376.

[**54**] Sacks HS, Fain JN, Bahouth SW, Ojha S, Frontini A, Budge H *et al.* Adult epicardial fat exhibits beige features. *J Clin Endocrinol Metab.* 2013; **98:** E1448-1455.

[**55**] van den Beukel JC, Grefhorst A, Hoogduijn MJ, Steenbergen J, Mastroberardino PG, Dor FJ *et al.* Women have more potential to induce browning of perirenal adipose tissue than men. *Obesity (Silver Spring).* 2015; **23:** 1671-1679.

[**56**] Rockstroh D, Landgraf K, Wagner IV, Gesing J, Tauscher R, Lakowa N *et al.* Direct evidence of brown adipocytes in different fat depots in children. *PLoS One.* 2015; **10:** e0117841.

[**57**] Sharp LZ, Shinoda K, Ohno H, Scheel, DW, Tomoda E, Ruiz L *et al.* Human BAT possesses molecular signatures that resemble beige/brite cells. *PLoS One.* 2012; **7:** e49452.

[**58**] Imran KM, Rahman N, Yoon D, Jeon M, Lee BT, Kim YS. Cryptotanshinone promotes commitment to the brown adipocyte lineage and mitochondrial biogenesis in C3H10T1/2 mesenchymal stem cells via AMPK and p38-MAPK signaling. *Biochim Biophys Acta.* 2017; **1862:** 1110-1120.

[**59**] Rogers NH, Landa A, Park S, Smith RG. Aging leads to a programmed loss of brown adipocytes in murine subcutaneous white adipose tissue. *Aging Cell.* 2012; **11:** 1074-1083.

[**60**] Khanh VC, Zulkifli AF, Tokunaga C, Yamashita T, Hiramatsu Y, Ohneda O. Aging impairs beige adipocyte differentiation of mesenchymal stem cells via the reduced expression of Sirtuin 1. *Biochem Biophys Res Commun.* 2018; **500:** 682-690.
