# Supplementary figures and images for: Identification of distinct transcriptome signatures of human adipose tissue from fifteen depots
Source: Eur J Hum Genet. 2020 Jul 13;28(12):1714–25. doi: 10.1038/s41431-020-0681-1 (PMC7784683; doi:10.1038/s41431-020-0681-1)

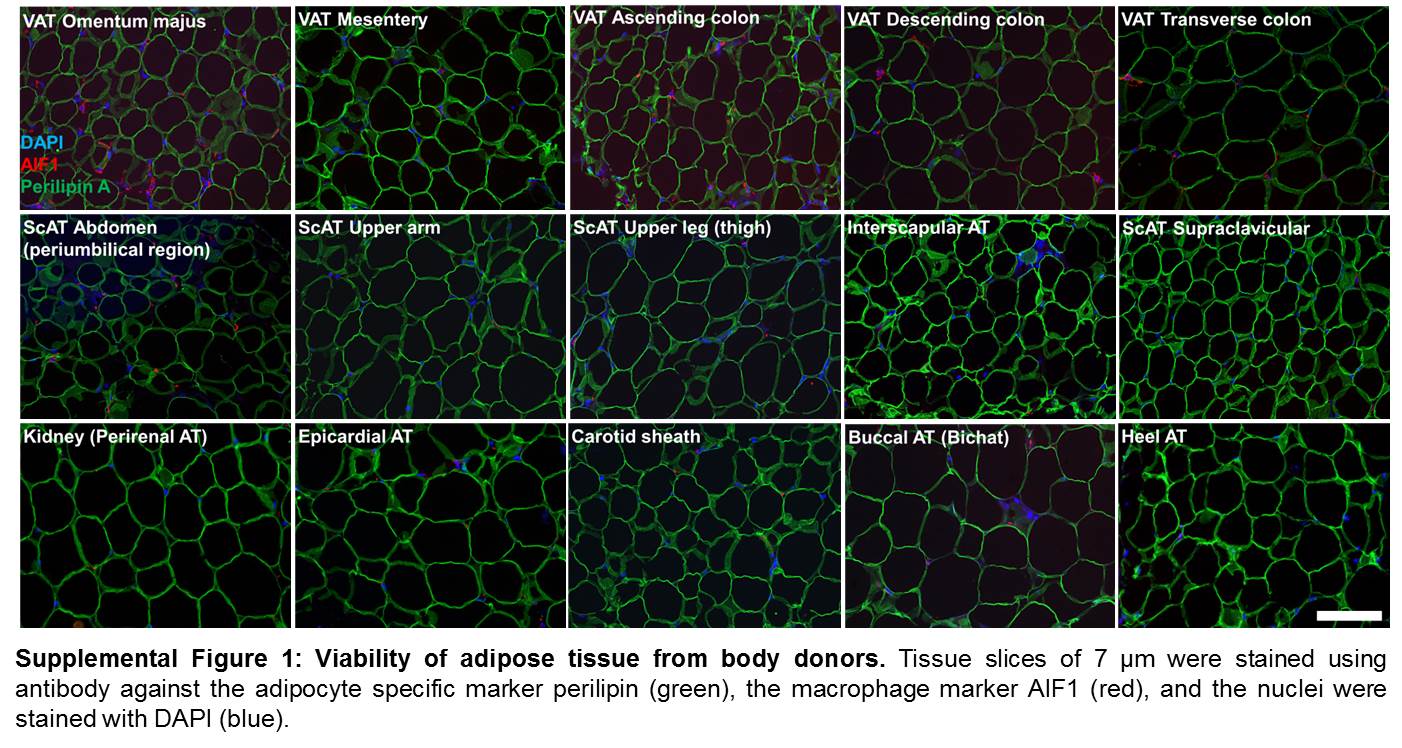

Supplement: Supplementary file 2 — Supplemental Figure 1: Viablility of AT from body donors. Tissue slices of 7 µm were stained using antibody against the adipocyte specific marker perilipin (green), the macrophage marker AIF1 (red), and the nuclei were stained with DAPI (blue). Scale bar represents 50 µm. [file 41431_2020_681_MOESM2_ESM.jpg]

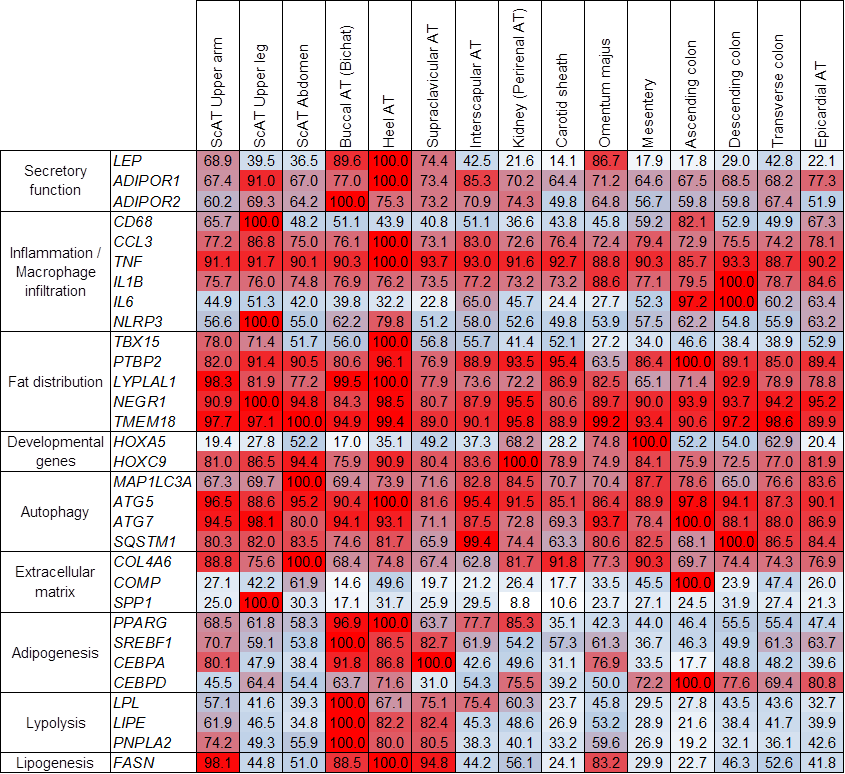

Supplement: Supplementary file 3 — Supplemental Figure 2: Proportion of gene expression between the tissues of selected biological functions and pathways. Calculation in percent based on the mean tissue expression of two body donors with a post mortem delay of 10 hours. White-blue-red coding=0-100%. (For readability, please open the figure with other software than Wndows photo.) [file 41431_2020_681_MOESM3_ESM.png]

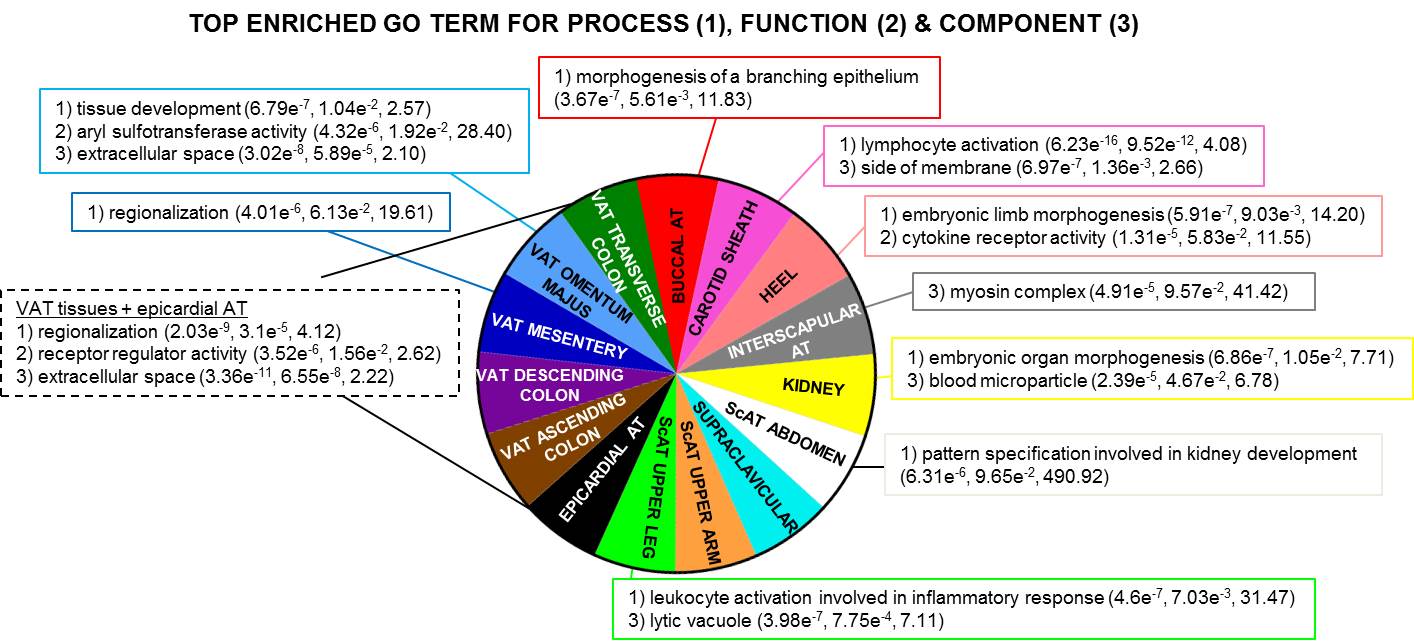

Supplement: Supplementary file 4 — Supplemental Figure 3: Top enriched GO term for process (1), function (2) & component (3). Boxes for the respective tissue display top enriched GO term with p<0.05 and false discovery rate (FDR) q-value<0.2. 1) process (p-value, FDR q-value, enrichment) and/or 2) function (p-value, FDR q-value, enrichment) and/or 3) component (p-value, FDR q-value, enrichment). Gene lists from the single tissue comparison against all other tissues from the visceral area including epicardial AT were also combined for GOrilla as the single information for these tissues would not withstand statistical significance with the respective cut off for the false discovery rate (dashed line box). Analyzed transcripts for this figure are given in “Gene Lists for GOrilla” in the Variable Lists collection V1-6 Supplemental document 1. [file 41431_2020_681_MOESM4_ESM.jpg]
