# Supplementary material for: Identification of distinct transcriptome signatures of human adipose tissue from fifteen depots
Source: Eur J Hum Genet. 2020 Jul 13;28(12):1714–25. doi: 10.1038/s41431-020-0681-1 (PMC7784683; doi:10.1038/s41431-020-0681-1)
Supplement: Supplementary file 5 — Supplemental Figure 4: Markers for beiging/browning. Given are the mRNA expression values for each sample represented by a dot for the following markers: BMP7, CIDEA, EBF2, FGF21, LHX8, PPARG, PRDM16, TBX1, TMEM26, UCP1. The continuous line (red) represents the mean over all samples. The small lines (green) represent the mean for all samples from one tissue location. The data displayed here are pre-processed, log2 transformed and are adjusted for post mortem delay, Sentrix barcode, age and sex. Of note, the Y-axes have different scaling. Further investigated marker with similar results (data not shown): ADAM17, BMP4, CAR4, TNFRSF9, CITED1, COX4, HOXC8/9, HSPB7, MIR133B, miR26-family, PPARGC1A, ZIC1. [file 41431_2020_681_MOESM5_ESM.docx]

|  |  |
| --- | --- |
|  |  |
|  |  |
|  |  |
|  |  |
| **Supplemental Figure 3: Markers for beiging/browning.** Given are the mRNA expression values for each sample represented by a dot for the following markers: *BMP7*, *CIDEA*, *EBF2*, *FGF21*, *LHX8*, *PPARG*, *PRDM16*, *TBX1*, *TMEM26*, *UCP1*. The continuous line (red) represents the mean over all samples. The small lines (green) represent the mean for all samples from one tissue location. The data displayed here are pre-processed, log_2_ transformed and are adjusted for post mortem delay, Sentrix barcode, age and sex. Of note, the Y-axes have different scaling. Further investigated markers with similar results (data not shown): *ADAM17*, *BMP4*, *CAR4*, *TNFRSF9*, *CITED1*, *COX4*, *HOXC8/9*, *HSPB7*, *MIR133B*, *miR26-family*, *PPARGC1A*, *ZIC1*. | |
